# One health in our backyard: Design and evaluation of an experiential learning experience for veterinary medical students

**DOI:** 10.1016/j.onehlt.2018.05.001

**Published:** 2018-05-02

**Authors:** Siobhan M. Mor, Jacqueline M. Norris, Katrina L. Bosward, Jenny-Ann L.M.L. Toribio, Michael P. Ward, Jaime Gongora, Meg Vost, Peter C. Higgins, Paul D. McGreevy, Peter J. White, Sanaa Zaki

**Affiliations:** a The University of Sydney, Faculty of Science, School of Veterinary Science, NSW 2006, Australia; b The University of Sydney, Marie Bashir Institute for Infectious Diseases and Biosecurity, NSW 2006, Australia

**Keywords:** One health, Field trip, Experiential learning, Veterinary education, Cultural competence, Zoonoses, Animal behavior, CPE, Centennial Parklands Experience, CPEC, Centennial Parklands Equestrian Centre, DVM, Doctor of Veterinary Medicine, IPE, Interprofessional Experience, MPH, Masters of Public Health

## Abstract

**Background:**

New educational approaches are needed to improve student understanding of the wider sociological and ecological determinants of health as well as professional responsibilities in related areas. Field trips allow students to observe interaction between plant, animal and human communities, making them an ideal tool for teaching One Health concepts.

**Methods:**

Veterinary medical students participated in a field trip to a local parklands area, frequented by humans, dogs, horses, and wildlife. Students rotated through 5 learning activities (‘stations’) that focused on: (1) response to exotic animal disease incursion (equine influenza); (2) impact of cultures and belief systems on professional practice; (3) management of dangerous dogs; (4) land use change, biodiversity and emerging infectious disease; and (5) management of environmentally-acquired zoonoses (botulism). Intended learning outcomes were for students to: evaluate the various roles and responsibilities of veterinarians in society; compare the benefits and risks associated with human-animal and animal-animal interactions; and evaluate the contributions made by various professionals in safeguarding the health and welfare of animals, humans and the environment. Following the field trip, students participated in a debrief exercise and completed an online survey on their experiences.

**Results:**

Feedback from students collected in 2016/2017 (n = 211) was overwhelmingly positive. The learning experience at each station was rated as 4 (‘Good’) or 5 (‘Very Good’) out of 5 by 82–96% of students. Responses to closed- and open-ended questions − as well as outputs generated in the debrief session − indicated that students achieved the learning outcomes. Overall, 94% of students agreed or strongly agreed that they had a better understanding of One Health because of the field trip.

**Conclusions:**

Field trips to local parklands are effective in promoting learning about One Health and can be incorporated into the core curriculum to maximize student exposure at relatively low cost.

## 1 Introduction

Following decades of improvements to population health in many countries, the world is faced with a number of formidable threats to global health, including antimicrobial resistance, emerging infectious diseases, food security, biodiversity loss and climate change [[1], [2], [3], [4]]. Many of these problems reflect the complex interplay between human, animal and environmental health and their solutions lie in collaborative approaches that draw on expertise from a wide range of disciplines (so-called “One Health”; [5]). Within the health professions, some have questioned whether modern curricula are equipping medical and veterinary medical graduates to tackle these challenges [6,7], arguing that the narrow technical focus of these programs is producing graduates with a limited understanding of the wider sociological and ecological contexts of population ill-health [6,8].

To be effective leaders in these areas, medical practitioners and veterinarians must have an appreciation for the interdependency of human, animal and environmental health (systems thinking) as well as the social, political, legal and cultural environments in which they work [9,10]. They also need to be able to assess and manage interactions between humans, animals and their environment [11] and work in multidisciplinary teams with an understanding of and appreciation for the different roles and responsibilities of various stakeholders [9]. While there have been numerous calls to reform medical [6,12] and veterinary medical [7,13,14] curricula to incorporate these core competencies, there are relatively few accounts in the literature of how this can be achieved.

Various models have been proposed and/or adopted to address these curricular deficiencies. Some universities have established dual degree programs (e.g. DVM/MPH; [15,16]) and stand-alone Masters programs focusing on One Health (e.g. University of Edinburgh, University of London). While these initiatives are important, they place increased demands on students and enroll fairly small numbers, making them less impactful at the professional level. Others have proposed or established opportunities for multidisciplinary interaction through the core curriculum – such as through taking common (pre-clinical) coursework [8,17] or participating in interprofessional experiences (IPE) [[18], [19], [20]]. These require considerable coordination across programs and, at many universities, may not be easily achievable in the short term, given time, logistic and geographic constraints. Further, and as noted by Courtenay et al. [20], most of the IPE initiatives described to date have focused on improving human patient care through enhanced understanding of the social determinants of health, with the role of animals and the physical environment rarely considered in these initiatives. Indeed, environmental science training is often lacking entirely from medical and veterinary medical curricula [8].

Field trips – which we define here as an off-campus visit under the supervision of one or more faculty member(s) – are common in natural science and anthropology curricula and allow students to observe interactions between plant, animal and human communities in their natural setting [21]. Educational goals of field trips vary but can include: promoting application and consolidation of classroom learning; deepening conceptual development; encouraging group interaction (both teacher, student and peer); and stimulating appreciation for and valuing of the environment [21,22]. We therefore believe they are a suitable model for teaching One Health. However, field trips are uncommon in clinical degree programs. Some universities have established summer institutes [23,24] and field school programs [25] which combine didactic learning with an international immersion experience in a developing country, as a means to promote learning in cultural competency and One Health. Such experiences may offer similar benefits to field trips but they are costly and often available to only a limited number of students. Further, while international experience is essential for addressing global health challenges, it is equally important to design local educational experiences, to promote equity and engagement by all students, as well as stimulate thinking about One Health issues in the more immediate vicinity.

In this paper we describe the design and evaluation of a One Health field trip undertaken by all veterinary medical students in the first week of their professional degree at the University of Sydney. Uniquely, this activity capitalizes on public parklands close to the main campus. The intended learning outcomes of the field trip were for students to:

1. Evaluate the various roles and responsibilities of veterinarians in society;
2. Compare the benefits and risks associated with human-animal and animal-animal interactions;
3. Evaluate the contributions made by various professionals, including veterinarians, in safeguarding the health and welfare of animals, humans and the environment.

## 2 Material and methods

### 2.1 Setting

The Doctor of Veterinary Medicine (DVM) at the University of Sydney is a 4-year graduate degree program introduced in 2015. Around 60% of the students enter the DVM via the combined Bachelor of Veterinary Biology/DVM program (6 years duration). During the planning phase for the degree One Health was identified by faculty members as an essential underlying theme for the DVM. Introduction of core One Health concepts at the beginning of the degree was therefore deemed a priority, so as to create a scaffold and shape the lens through which students engaged with their veterinary medical training.

The Centennial Parklands Experience (CPE) is a full-day field trip which takes place at the end of the first week of the DVM (February each year). Accessible by public transport, the Centennial Parklands are located in eastern Sydney, approximately 3.5 km from Sydney's Central Business District and 4.5 km from the University. The Parklands encompass three connecting public parks: Centennial Park, Moore Park and Queen's Park. Centennial Park comprises open space, lightly wooded areas, swamp and ponds set over an area of 2.2 sq km. It was officially opened in 1888, marking 100 years since European settlement.

Today, Centennial Park is used by many animals that visit (humans, dogs, horses) or live (waterfowl, fruit bats [“flying-foxes”]) in the park, which is rich with flora and waterways. The park is an important part of the social landscape of Sydney, with people of diverse cultural backgrounds using the space for a variety of reasons (e.g. fitness and recreation, celebration of special occasions); an estimated 200 million visits take place each year. Like any ecosystem there is potential for interactions between humans, domestic and wild animals, and the surrounding environment. Traffic principally occurs along a 3.8 km circular road (Grand Drive) that passes through the park and has dedicated lanes for car drivers, cyclists, pedestrians and horse riders. Off-leash walking of dogs is permitted in designated areas. Equestrian facilities in the Park include the riding track that loops through the park alongside the Grand Drive, as well as dressage and show-jumping arenas. These are used by both commercial operators (e.g. park rides, riding lessons) and private horse owners. Horses are stabled in the Centennial Parklands Equestrian Centre (CPEC), which is set within the adjacent Moore Park.

### 2.2 Learning activities and logistics

Students are allocated into ten groups (~12–14 students each) and rotate through activities at five stations (described below) which are strategically placed within the Parklands. Given that students are only in the first week of their veterinary medical degree program, activities at each station are designed to drawn on common knowledge and provide only the minimal information needed for students to engage with the discussion. Each activity lasts 45–50 min after which students walk, as a group, to the next station using the map provided (see Supplementary material). The CPE has been replicated each year since 2015 and is coordinated and facilitated by faculty members within the School of Veterinary Science. Each station has a minimum of two facilitators, enabling two groups to run simultaneously at each station. Additionally, two faculty members and an administrative staff member assist with logistical preparations and are available to address matters that arise on the day. Financial costs associated with the field trip (borne by the school) relate to purchase of stationary, lunch and once-off supplies (e.g. audio speakers, easels) (~AUD600 annually). Students are encouraged to use public transport and bring items for personal use (e.g. sunscreen, towel to sit on).

### 2.3 Station 1: Equestrian Centre (equine influenza)

The learning focus at this station is the role of the veterinary medical profession in relation to disease control, including the legal obligation to report notifiable diseases (developed by JAT). The station is located within CPEC which includes several large stable pavilions (accommodating up to 200 horses), a veterinary clinic, riding arenas, lunging and grooming facilities. CPEC was the site of an equine influenza (EI) outbreak in August 2007. On arrival students are invited to explore CPEC to observe human-animal and animal-animal interactions and identify opportunities for infectious disease spread. Students then discuss a disease outbreak scenario, imagining they are a veterinarian employed at the CPEC veterinary clinic. A scenario of coughing horses observed over 4 days at CPEC is read out by the facilitator and a volunteer student records case numbers and pavilion locations as the outbreak scenario evolves (97 cases out of 160 horses by Day 4). Students then participate in a facilitated discussion about the type of disease process, modes of transmission, and the time point at which they would consider that something unusual was happening that might need to be reported to government authorities. Students are then provided with additional information, namely that: (1) veterinarians were notified of an EI outbreak amongst shuttle stallions at the Quarantine Station in western Sydney three days before the first coughing horse appeared at CPEC; and (2) EI vaccination of horses is not practiced in Australia because it is an exotic disease. Students are given an opportunity to re-consider the timing of reporting, and to propose appropriate disease control actions and communication with CPEC management, businesses and horse owners in their implementation. To close, a summary is read of the actual EI outbreak at CPEC, which was the first infected premise identified outside the quarantine station. The summary statement includes comments reflecting the varying opinions amongst veterinarians and CPEC management regarding notifying the state government authority about a suspect exotic disease outbreak at the premise [26,27].

### 2.4 Station 2: Paperbark Grove (cultural competency)

The learning focus at this station is the impact of cultures and belief systems on professional (veterinary) practice, and how these influence different perceptions of animals (developed by JG and MV). In various years, this station has been facilitated by an Australian Indigenous person, a biologist, an anthropologist and a community development specialist. Facilitators begin by acknowledging the traditional owners of the land. They mention the contributions that Indigenous peoples made, and continue to make, to caring for country as well as the cultural activities, management and conservation practices that would have occurred in the space that is now Centennial Parklands, prior to European arrival. This includes discussion of the animals and plants that were available to Indigenous peoples and the engravings and artefacts found near the parklands describing native animals and human activity. These reflections are used to highlight the relevance to veterinarians of learning about cultural aspects related to the place in which their professional practice takes place. Students are invited to contribute if they have knowledge about these areas. Following this, a brief overview of foundational cultural competence concepts – including the diverse definitions and complexity of ‘culture’ – is presented to frame the subsequent discussions. A worksheet is used to guide students through a number of self-reflections, including on their own cultural perceptions of animals and the role that animals play, their motivations for wanting to work with animals, and their perceived role of animals in a culture that is different from their own. Subsequently, students listen to audio recordings with perspectives from various cultural backgrounds including by those of Middle Eastern, African, Asian and Aboriginal Australian descent. Working in small groups, students are then asked to reflect on the similarities, intersections and differences between their own perceptions and that of different or diverse cultures and what the impact may be on their professional practice when cultural factors are considered. Further classroom-based activities take place the following week and are designed to deepen student reflections on cultural competence in client interactions.

### 2.5 Station 3: Sandstone Ridge (dog bite)

The learning focus at this station is the societal role of veterinarians within the construct of responsible pet ownership and, in particular, their role in pet management issues (developed by PH). The station is physically located in the leash-free area of Centennial Park where dogs can be observed interacting with each other and their owners. To start, the facilitators describe a scenario in which a male, undesexed dog (“Bluey”) bites another dog (“Charlie”) whilst playing in the park. Charlie's owner is also bitten as she tries to free her dog. She subsequently informs the local council and is taking legal action over the incident. Students are invited to assume the role of a veterinarian at a local veterinary clinic. Bluey's owner is seeking advice and clarification of his legal position after receiving a letter stating that Bluey could be classified as a dangerous dog and potentially seized. Students are then led though a workshop on the benefits of regular walking with a dog, the responsibilities of owners when they walk their dog in a public space and how control of a dog may be defined and achieved. They also reflect on the physical, psychological and behavioural consequences of dog bite, to both human and canine victims, as well as management of injuries. Examining the issues through a legal lens, students are asked to consider whether the owner might be required to euthanize Bluey. They are asked to speculate on how the situation would change had the incident unfolded on private property rather than at the park. This discussion leads to an exploration of the local legal definition of a “dangerous dog” and the requirements for keeping a dangerous dog. Finally, the students are asked to determine whether the owner could be charged by the police or rangers, and what their legal responsibility is, as the attending veterinarian. They are then asked to consider the roles and responsibilities of attending veterinarians who have been subpoenaed as expert witnesses. The discussion ends with the students summarizing their recommendations to Bluey's owner.

### 2.6 Station 4: Lachlan Swamp (bat relocation)

**Fig. 1:**
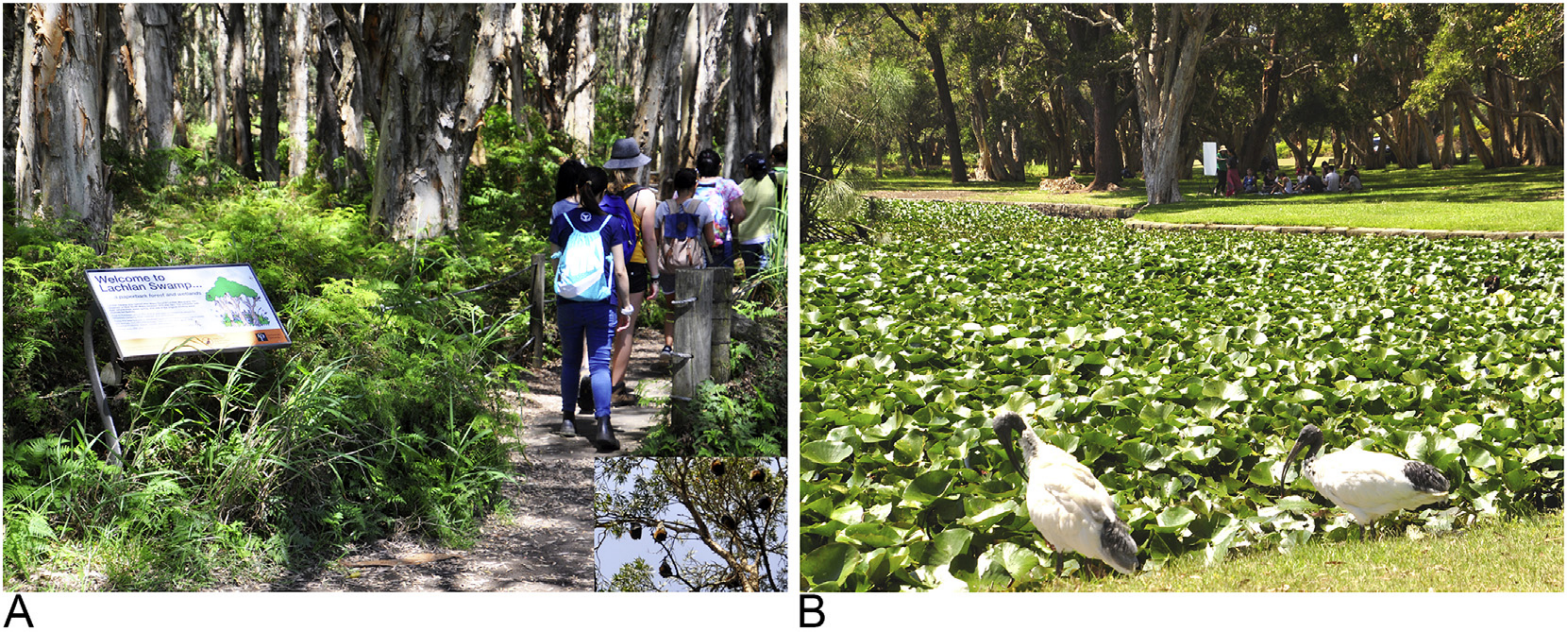
Photographs taken on-site during the Centennial Parklands Experience, 2016. A, Students walking from Station 4 (Bat relocation) into Lachlan Swamp as flying-foxes hang over (inset); B, View across Lily Pond (foreground) to Station 5 (Botulism) where students can be seen discussing the case with the facilitators (background). Credit: Anke Wiethoelter.

The learning focus at this station is the impact of human-induced land use changes on biodiversity and its contribution to emerging infectious diseases (developed by SM). The station is situated adjacent to Lachlan Swamp, which is the roosting site for large colony of grey-headed flying-foxes (*Pteropus poliocephalus*; numbering between 5000 and 45,000, depending on season and year; Fig. 1A). At this station, students discuss a (real) legal case in which the Royal Botanic Gardens, located 4 km north of the Centennial Parklands, sought permission to disperse a large flying-fox colony that was damaging historically important tree specimens. Four volunteers present excerpts adapted from the legal testimony and other public documents. This includes statements by the Royal Botanic Gardens and Domain Trust (in favor of relocation) and Bat Advocacy NSW (non-for-profit group; opposes relocation), as well as two expert witnesses, a wildlife ecologist and a veterinary virologist. Remaining students serve as jury members; students write their names on a sticky note and are invited to place their name on a board indicating whether or not they are in favor of the relocation. Students are invited to share their views on why the colony should/should not be dispersed and explain if/why their view changed after listening to the testimonials. During the discussion, the facilitator asks probing questions to encourage deeper thinking about the causes of the problem. Students learn that *P. poliocephalus* is threatened and in decline because of habitat disruption caused by deforestation and agriculture. They also learn that other human activities, such as planting of fig trees (*Ficus* spp.) alongside roads, have pulled these wild animals into urban landscapes, as natural food reserves have become depleted. It is eventually revealed that the dispersal went ahead and many of the flying-foxes re-settled in Lachlan Swamp. Students are invited to walk along the boardwalk beneath the colony as they reflect on the new risks that might arise as a result of the bats being in this location. The activity concludes with a discussion of locally-important zoonotic diseases that come from flying-foxes (Hendra virus and Australian bat lyssavirus) and possible risks/concerns to humans, horses and dogs that use the park.

### 2.7 Station 5: Lily Pond (botulism)

The learning focus at this station is the contribution of ecosystem health to human and animal health and well-being, as exemplified by the disease, botulism (developed by KB and JN). The Parklands have been the site of previous botulism outbreaks involving waterfowl and dogs; this has required alterations to and constant monitoring of the lake ecosystems. The station is located between three large ponds (Fig. 1B). Initially, students work with facilitators to discuss and come to a consensus regarding their collective, pre-existing knowledge of botulism (and botox), including risk factors and potential clinical outcomes in different species. Students then explore their immediate environment in smaller groups to examine the apparent health of the lakes, identify potential risk factors, and note the range of animal species (including humans) that come to share and interact in this space. Following a discussion of the groups' findings regarding the immediate environment, the students are invited to assume the role of a local veterinary practitioner that is presented with a Pacific Black Duck (*Anas superciliosa*) exhibiting signs consistent with botulism. Students are asked to consider more broadly the other professionals they would engage to assist them in managing this environmentally-acquired disease. They listen to short written testimonies from an Environmental Officer at Centennial Parklands, a medical practitioner specializing in infectious diseases and a local veterinarian and thereby explore the valuable insights provided by these colleagues. Through this exercise students extend or confirm their understanding of the causes of botulism and its risk factors and explore the importance of considering the broad ecosystem in the etiology of disease. They also learn the importance and value in communicating broadly with other professionals.

### 2.8 Debrief

**Fig. 2:**
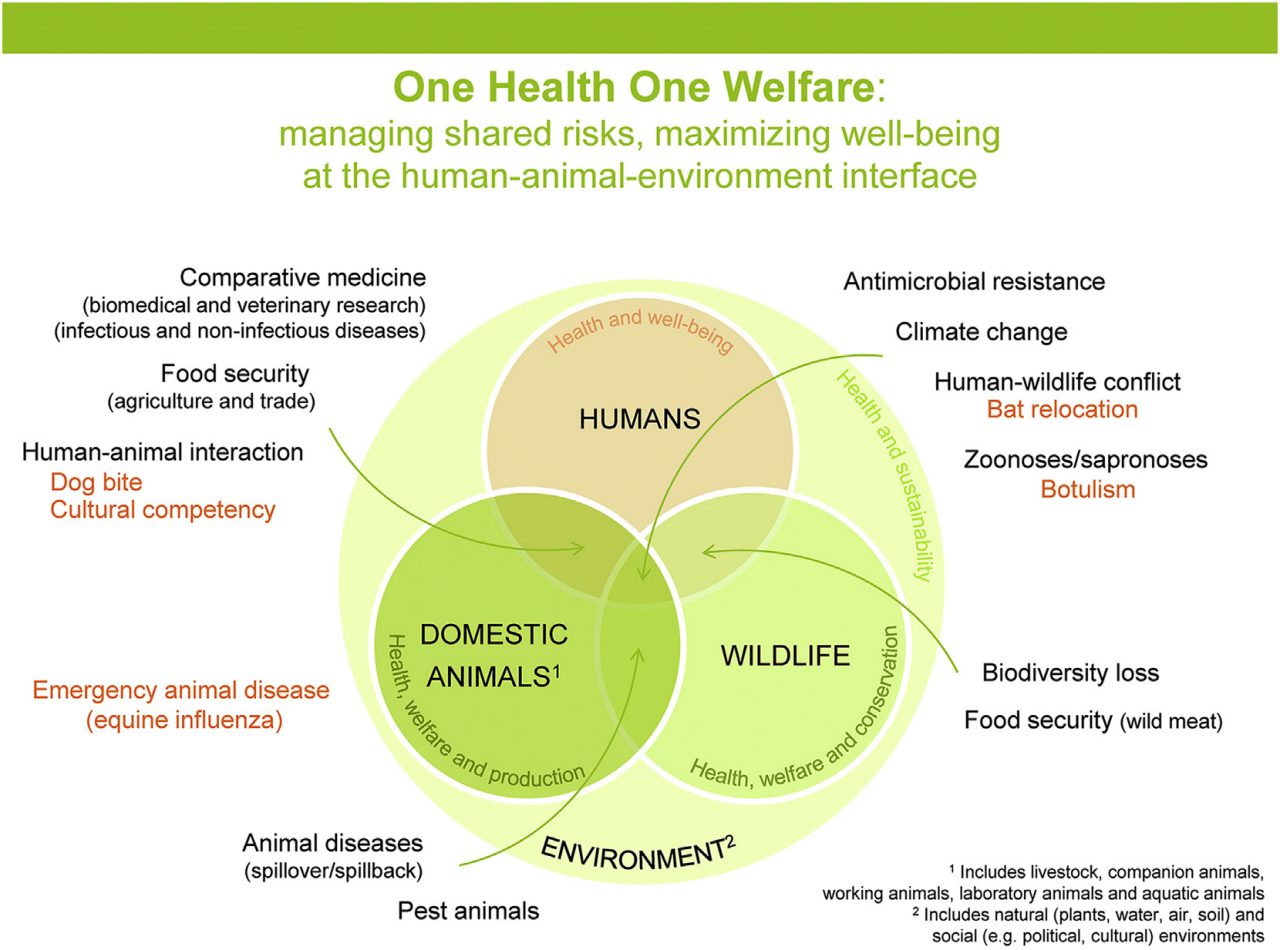
Venn diagram used during the debrief session for the Centennial Parklands Experience. The diagram aims to put each station within the context of a broader range of One Health issues.

Within one week of the field trip, students meet with the lead facilitator (SM) to debrief the learning experience. Students are provided with a brief introduction to the concept and origins of One Health (and related terms such as Planetary Health, Ecohealth and One Medicine). A Venn diagram (Fig. 2) is used to frame the topics discussed at each station within the context of a broader range of One Health issues. Students then work in groups to develop online Wikis documenting their learning on the roles and responsibilities of veterinarians in society, the risks and benefits associated with human-animal and animal-animal interactions and the multi-disciplinary collaborations needed to manage problems discussed during the CPE (Table 1). In this way, students are encouraged to reflect on and integrate learning across the different stations.

**Fig. 3:**
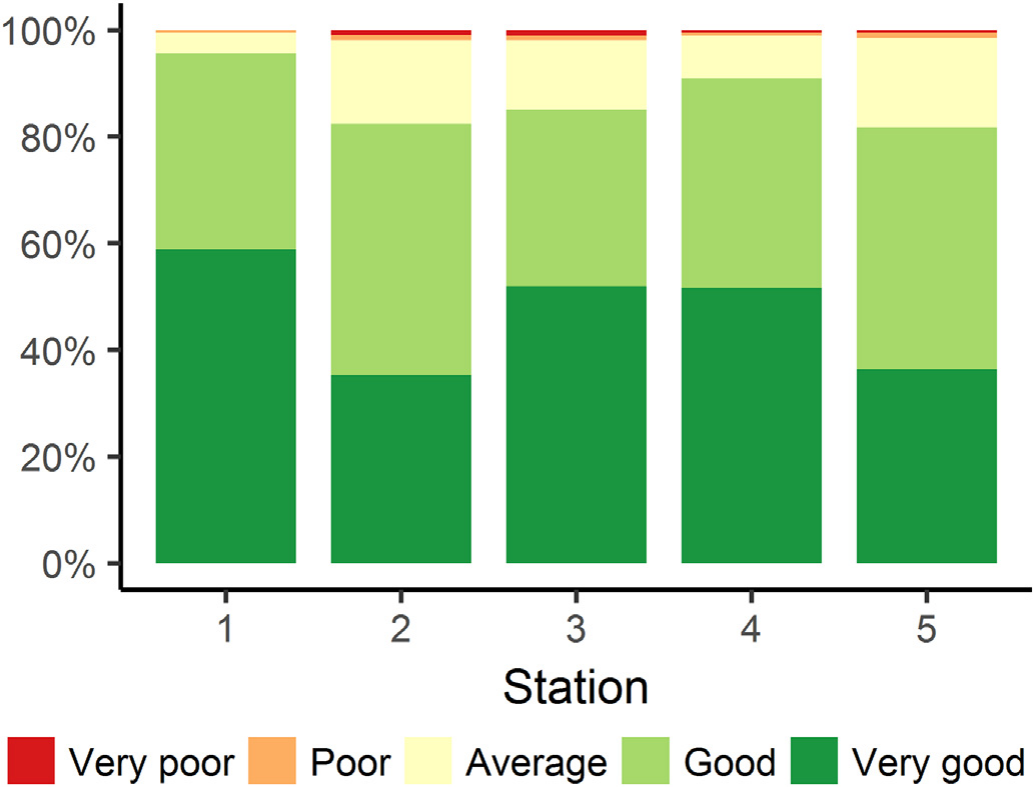
Frequency distributions of veterinary medical student ratings of the learning experience at each station at the Centennial Parklands Experience, 2015/2016. Station 1 = Equine influenza; 2 = Cultural competency; 3 = Dog bite; 4 = Bat relocation; 5 = Botulism.

**Table 1:** Example of a table produced by veterinary medical students for the Wiki during the debrief session for the Centennial Parklands Experience. The table has been minimally edited for clarity (edits shown in square parentheses). Facilitator prompts are indicated in the footnotes and are related to the learning outcomes.

Station	Roles and responsibilities [of veterinarians]^a^	Risks [associated with human-animal and animal-animal interaction]^b^	Benefits [associated with human-animal and animal-animal interaction]^b^	Collaborators^c^
Equine influenza	Examine horses from an unbiased perspective Communication with other vets, stables, and horse owners Report disease Maintain quarantine standards - proper [personal protective equipment]	Animal to animal transmission	Awareness Managed to beat and eradicate foreign disease – protocols work	Virologists Veterinary specialists [State government] Horse owners Stable managers
Cultural competency	Aware of others cultures Use best judgment when presenting a client with options Consider the different roles of animals within various cultures	Offending people	Expand your cultural competence	Clients Cultural spokespersons
Dog bite	Educate and inform owners on proper behaviour and training techniques Inform clients on local laws Diffuse stressful situations Suggest solutions for clients once an incident has happened Obligation to be ambassadors to the public about prevention of dog bites	Breed stereotypes Costs to clients Risk of being bitten Euthanasia	Preventing further dog bites	Local governments and councils Park rangers Paramedics and doctors Lawyers Animal trainers
Bat relocation	Obligation to the preservation of wildlife Educate on techniques used for [relocation]	Zoonotic disease Risks to the bat population Financial costs Potential increase in transmission of zoonotic disease	Save trees Potential decrease in transmission of zoonotic disease	Activists Ecologists Virologists Local governments and councils Park management Public
Botulism	Awareness and reporting on zoonotic [diseases]	Causes [disease] in humans	Awareness	Environmental officer Small animal [veterinarian] Clinical pathologist Microbiologist Doctors

a Facilitator prompt: What roles and responsibilities of veterinarians in society were highlighted at each station? Others?

b Facilitator prompt: What benefits and risks of human-animal and animal-animal interaction were highlighted at each station? Others?

c Facilitator prompt: What professionals also contributed knowledge/expertise to the scenario at each station? Who else do veterinarians collaborate with?

### 2.9 Student survey

In the remaining 10 min of the debrief sessions held in 2016 and 2017, students were invited to provide feedback on the CPE via an online survey (ethics approval number 2016/687). The survey was implemented in Survey Monkey and included a series of closed (n = 10) and open-ended (n = 2) questions (see Supplementary materials). The survey asked students about their previous exposure to the term “One Health”, and used 5-point Likert scales to invite students to rate their experience at each station. Students were also asked the extent to which the CPE helped them to achieve each learning outcome and improve their understanding of One Health. They were also invited to share their thoughts on the best aspects of the CPE. At the end of the survey, students were asked if they gave their permission for their anonymous responses to be included in a publication.

### 2.10 Analysis

Data from questions that had a binary response were summarized using proportions, disaggregated by year. Data from Likert scale questions were examined using frequency distributions. The proportions of students selecting 4 or 5 out of 5 were examined separately for each year. These categories were collapsed into a binary variable (<4, ≥4) for analysis. Chi-square tests were used to test whether responses differed between years and with previous exposure to the term “One Health”. No significant differences were observed between years and thus proportions were pooled across years for reporting purposes. Ratings for each station were also pooled across years and plotted using R statistical software version 3.1.3 (R Foundation for Statistical Computing, Vienna, Austria). Responses to open-ended questions were read and re-read by one of the authors (SM) to identify themes emergent from the data as well as to identify illustrative quotes.

## 3 Results

Feedback was collected from 217/281 students who participated in the CPE in 2016 and 2017 (response rate = 77%). Two-hundred and eleven students gave permission to have their feedback included in this publication (121/123 in 2016, 90/94 in 2017). The data from one student who was enrolled in 2016 were excluded because that student did not attend the field trip and thus provided responses that were neutral on most questions.

Fifty-nine per cent of students indicated that they had heard the term “One Health” before participating in the CPE (60% in 2016, 57% in 2017). Frequency distributions of student ratings of the learning experience at each station are shown in Fig. 3. Depending on the station, between 82% and 96% of students rated the learning experience as ‘Good’ or ‘Very good’. For all stations except cultural competency, there were no significant differences between the ratings of stations based on previous exposure to the term “One Health”. In contrast, students that had previous exposure to the term were more likely to rate the cultural competency station ≥ 4 out of 5 (87% vs 76%; p = 0.04).

In regards to achieving the learning outcomes, the majority of students agreed (A) or strongly agreed (SA) that the CPE improved their understanding of: the roles and responsibilities of veterinarians in society (63% A and 30% SA); the benefits and risks associated with human and animal interactions (64% A and 25% SA); and the contributions made by various professionals in safeguarding the health and welfare of animals, humans and the environment (63% A and 31% SA). No more than two students in any one year disagreed with these statements and none strongly disagreed in either year. There were no significant differences in levels of agreement based on previous exposure to the term “One Health”, with regard to learning outcomes.

Themes emerging from open-ended comments in response to the question “What was the best part about the CPE” (n = 210) included student learning, the learning environment, and appreciation for specific stations and organizational aspects (Table 2). Students clearly appreciated the outdoor learning environment, with many noting that it was good to be outside of the classroom. The interactive and discussion-based nature of the activities, as well as the diversity and real-life nature of the scenarios, were also positive highlights. Embedding scenarios in the park was specifically commended, with one student stating “*This exercise was beautifully married to [Centennial Park]. Although the content was excellent, it would not have had the same lasting impact if it were presented in the classroom.*” Station 1 (equine influenza) and Station 3 (dog bite) proved most popular in open-ended comments; students appreciated the opportunity to observe animals whilst discussing more technical aspects related to these stations. These stations also had amongst the highest proportion of students reporting that the learning experience was ‘Good’ or ‘Very good’ (96% and 85%, respectively; Fig. 3). Reasons offered for ‘Poor’ or ‘Very poor’ ratings of stations included: previous experience/simplicity of the scenarios (dog bite and cultural competency; n = 3), perceived irrelevance/limited reference to veterinarians (cultural competency and botulism; n = 2); difficulty hearing discussion (dog bite and botulism; n = 2); and limited background knowledge to engage with scenario (botulism; n = 1).

**Table 2:** Selected feedback from veterinary medical students after attending the Centennial Parklands Experience. Students responded to the question: “What was the best part about the Centennial Parklands Experience.” Comments are organized by theme.

Theme	Student comment
Student learning	Loved the chance to experience real-life applications beyond just clinical work. Made me feel like my degree will be relevant in society. Expanding on preconceptions of what it is to be a vet. Learning about the different interactions that I didn't even know existed between animal-human or animal-animal. Seeing practical examples of the role veterinarians play in the wider society and the complex web of interactions between vets and other people/professions. Understanding the importance of one health, understanding the diverse roles veterinarians have (public health or service). The scope of topics that involved the veterinary profession was a good way to open up my perspectives. The application of the different aspect of the veterinary profession in a relevant way with actual real cases instead of just telling us the different aspects, and the opportunity to think for ourselves about them.
Learning environment	Being in a setting that allowed visual contact with the ideas being expressed in the lessons and the small group activities that allowed discussion. Visiting each station to actually see and feel things that are related to the topics. The changing environment meant we were more stimulated and excited to learn. Learning outside, sometimes at the site of the event (eg. equine influenza) meant that I felt more connected to the learning experience. Getting to not only talk about the bigger picture of our profession but being able to see it at the park.
Appreciation for specific stations	I really enjoyed the dog bite scenario, it was very discussion based and was an area of interest for me and could very clearly see the applicability of those skills in real life especially with small animal vets. We also talked about preventative measures such as training and socialisation rather than just discussing how to address the situation after a dog bite has already occurred. It was especially great to be able to see it in a dog park where dog behaviour could be observed first hand while discussing it. I really liked the cultural session as it opened my eyes to more of what a vet is expected to fulfill in a society The botulism station with the dead duck really showed how easy it would be for a case of botulism to occur. Talking about actual disease outbreaks and how they are dealt with (ie. botulism and equine influenza) I enjoyed getting to see and learn about the flying foxes
Appreciation for organizational aspects	Meeting staff and learning their different role in association with the vet school. The opportunity to further bond with team members. Lunch.

Overall, 94% of students agreed or strongly agreed that they had a better understanding of One Health because of the CPE (63% A and 31% SA). This proportion was similar for students who had and had not been exposed to the term “One Health” prior to participating in the CPE (96% vs 93%, p = 0.82).

## 4 Discussion

In this paper we describe the incorporation of a One Health field trip into the core veterinary medical curriculum at the University of Sydney. Other universities have leveraged the local surroundings to expand medical and veterinary medical student awareness of social determinants of health. For instance, some have established medical and/or veterinary community clinics [20,28] where students work alongside trained professionals to provide clinical care to underserved communities. Further, one university implemented a community-guided bus trip through local neighborhoods to teach medical interns about challenges affecting their patients [29]. Similar to this, our veterinary medical students participate in community service programs in collaboration with an animal welfare organization during Years 2–4 of the DVM degree. Insights learned through evaluation of these initiatives confirm that these experiences greatly expand student understanding of the social determinants of human health [29] as well as the connection between human and animal welfare (One Welfare) [28]. Nevertheless, these experiences do not extend to consideration of the ecological factors that contribute to health and wellbeing outcomes.

The intended learning outcomes for the CPE were informed in part by the core competencies outlined by Frankson et al. [9] as well as graduate attributes and a road map of learning outcomes that was developed during the planning/design phase of the DVM. Learning activities developed for the CPE were selected to emphasize different topics relevant to One Health, such as zoonoses, environmental health, human-animal interaction and comparative pathology. Some consideration was given to having a station relating to foodborne illness (picnic), although this was not pursued for logistical reasons. Discussion and activities at all stations were directly linked to the immediate location within Centennial Parklands and have undergone minor modification since inception in 2015 in response to student feedback. To encourage systems thinking, facilitators drew connections between the broader issues being discussed in the park to scenarios seen in clinical practice and vice versa. For instance, the focus on coughing horses at Station 1 (equine influenza) leads to a discussion of the implications for disease control and eradication, and issues of maintaining Australia's status as an exporting country for international trade purposes. At Station 4 (bat relocation) the picture emerges that contact between bats, humans, dogs and horses – and associated risk of pathogen spillover – might be avoided if flying-fox habitat were preserved. Similarly, at Station 5 (botulism) the discussion of a case of botulism in a duck presenting to a local veterinary clinic emphasizes the need for veterinarians to view their patients in the context of the ecosystem in which they live.

Cultural competency (station 2) is one of six core qualities that are required to be developed by every graduate of the University of Sydney. This graduate attribute is introduced during the CPE and developed through the DVM using classroom teaching and other modalities (e.g. online learning, clinical communication skills laboratories) which enable students to explore various dimensions of cultural competence, including interactions with other professionals and disciplines. During the CPE, facilitators move students through reflections that culminate in a discussion of the impact of including considerations of culture in veterinary professional practice. Opening statements link the station to the Centennial Parklands setting by referring to previous usage of the land, plants and animals in that location by Indigenous peoples for fishing, hunting and ceremonial purposes. This helps students appreciate that the parklands were not always used in the way they are now (for recreational purposes), and that Indigenous communities managed and viewed the same land in a very different way, and continue to do so. Opening the station this way also moves students to a more reflective space, using the concepts of reflexive and transformative processes, by presenting the participants with an alternative way of perceiving the space around them [30]. We found students without prior exposure to the term “One Health” were less likely to rate this station as ‘Good’ or ‘Very good’. Possible reasons for this difference may be that students without such exposure are younger or have less real-life experience. Tension between cultural values and animal welfare were also noted with one student stating “*[I] still do not see the relevance of cultural competence to the degree. Welfare of the animal should be made a priority over cultural beliefs. Modern Australia has a liberal western value system, which we should accept and promote.*” (This student reported no prior exposure to One Health). In contrast, students that have been sensitized to One Health may be more willing to engage with the concepts discussed at this station. For example, one student stated “*I enjoyed the cultural competencies [sic] station and learning how different cultural backgrounds can lead to different medical treatments that they agree with*”. In sum, teaching One Health concepts and approaches may help students appreciate the relevance of cultural competency to professional veterinary practice. Conversely, field experiences in culturally diverse settings may help students better understand the One Health concept in a more holistic sense.

As an immersive learning experience, the CPE was designed to promote observation, reflection and conceptualization of One Health in a way that connects students with their local environment. This approach is consistent with Kolb's experiential learning theory which views learning as a “process whereby knowledge is created through transformation of experience” [31]. Introduction of the concepts of One Health in the first week of the degree provides a foundation and perspective for future learning that extends the focus from clinical to broader community perspectives and normalizes the culture of interprofessional collaboration. As with any evaluation based on self-report, there are several limitations to this study. In particular, student feedback – which was overwhelmingly positive – likely reflects both a sense of achievement of the learning outcomes as well as satisfaction and interest in the scenarios discussed at each station. Nonetheless, the contents of the Wikis provide evidence that students were able to evaluate and characterize the roles of veterinarians in society, the risks and benefits of human-animal and animal-animal interaction, and value of interprofessional collaboration, suggesting that the CPE does promote student learning in these areas. This was also reflected in the responses to open-ended questions and by facilitators' observations of student contributions to discussion on the day.

Limiting participation in CPE to veterinary medical students simplified the logistics of the field trip considerably, although this also meant there were no opportunities for direct engagement with other health professional students. Nonetheless, facilitation by veterinarians and animal scientists from a variety of disciplines (microbiology, veterinary epidemiology, public health, animal behaviour, urban animal management, animal welfare, and conservation biology) as well as explicit reflection on multidisciplinary collaboration in the debrief session cultivated an awareness of the need for different disciplines to work together to address complex health problems. This was evidenced in open-ended comments where one student, for example, stated that the best part was the CPE was “*One Heath Concepts [sic] and how we work together to improve life everywhere*.”

## 5 Conclusion

The Centennial Parklands Experience was designed to instill awareness in veterinary medical students at the University of Sydney of the concept of One Health and broader roles of veterinarians in a rapidly changing world where multidisciplinary collaboration will be essential to managing risks at the intersection of human, animal and environmental health. The CPE was well received by students, who reported that the experience improved their understanding of the roles and responsibilities of veterinarians, the risks and benefits associated with human-animal and animal-animal interaction and collaboration between veterinarians and others to address local issues that are germane to One Health. The field trip is an embedded part of the core curriculum and utilizes a local parklands area that is accessible by public transport. This approach means that a maximum number of students can participate at relatively low cost.

The following is the supplementary data related to this article.

**Supplementary Fig. 1:**
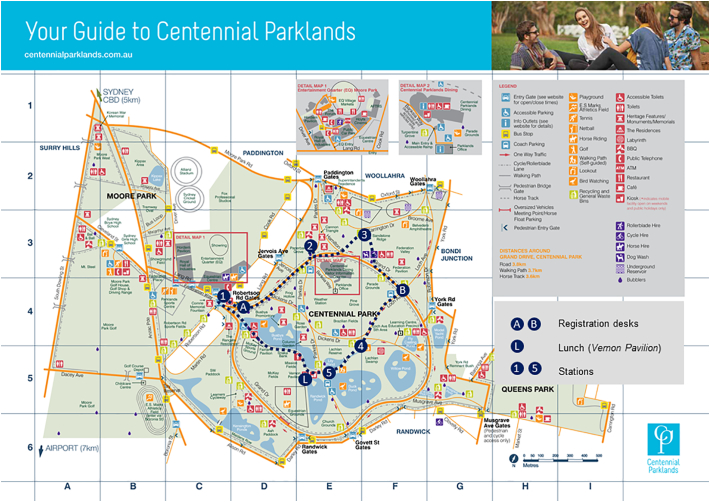
Map used by students for the Centennial Parklands Experience.

Supplementary materials Image 1

## Ethics approval and consent to participate

The project was reviewed and approved by the University of Sydney Human Research Ethics Committee (project no. 2016/687).

## Consent for publication

Students indicated their consent for publication at the end of the online survey.

## Availability of data and material

All data generated or analyzed during this study are included in this published article.

## Competing interests

The authors declare that they have no competing interests.

## Funding

We thank the Office of the Deputy Vice-Chancellor Indigenous Strategy and Services for providing financial support to JG for the cultural competence station. No other funding was obtained in support of this research.

## Authors' contributions

All authors contributed to the design of the CPE. SM came up with the initial concept for the CPE, designed the student evaluation survey, analyzed the data and wrote the initial draft of the manuscript. JAT, JG, MV, PH, PM, SM and JN contributed descriptions for learning activities for the manuscript. All authors provided comments on drafts and read and approved the final manuscript.
