# Supplementary material for: One health in our backyard: Design and evaluation of an experiential learning experience for veterinary medical students
Source: One Health. 2018 May 2;5:57–64. doi: 10.1016/j.onehlt.2018.05.001 (PMC6000817; doi:10.1016/j.onehlt.2018.05.001)
Supplement: Supplementary file 1 — Supplementary materials [file mmc1.pdf]

CP questionnaire

* 1. Had you heard of the term One Health (or similar term) before attending the Centennial Parklands (CP) Experience?

* 2. Learning experience

	Rating
How would you rate the learning experience at the Equestrian Centre (equine influenza)?	
How would you rate the learning experience at the Paperbark Grove station (cultural competency)?	
How would you rate the learning experience at the Federation Pavilion (dog bite)?	
How would you rate the learning experience at the Lachlan Swamp (bat relocation)?	
How would you rate the learning experience at the Vernon Pavilion (botulism)?	

Your feedback will help us make improvements for next year. If you selected Very Poor or Poor for any station(s), please explain why. Write N/A if you did not select Very Poor or Poor for any station

* 3. CP Experience

Overall, the CP Experience improved my understanding of the roles and responsibilities of veterinarians in society.	
Overall, the CP Experience improved my understanding of the benefits and risks associated with human and animal interactions.	
Overall, the CP Experience improved my understanding of the contributions made by various professionals in safeguarding the health and welfare of animals, humans and the environment.	

* 4. What was the best part about the CP Experience?

* 5. I have a better understanding of One Health because of the CP Experience.

* 6. The CP Experience is a unique learning experience not offered by any other university. We would therefore like to disseminate information about the experience in an educational journal. Do you give permission for your responses to be included (anonymously) in a future publication on the CP Experience?

7. If you are willing to be contacted about any of the information you provided here, please enter your email address. Write N/A if you do not agree to be contacted.
